# Our Contemporaries

**Published:** 1912-11

**Authors:** 


### OUR CONTEMPORARIES.

The *Continent* of April 18, emphasizes a fact that physicians somtimes overlook, through familiarity. Under the title of the Superlative Heroes, it pays an eloquent tribute to the courage displayed by patients.

The *Medical Council* will henceforth be continued under the business management of Mrs. J. J. Taylor, widow of the former editor and proprietor, with Dr. Thomas S. Blair long a member of the editorial staff, as editor. We have known Dr. Blair since his student days at the University of Michigan where he already began to show the ability which has characterized his maturer years.

The *Doctor's Factotum* has recently come to our desk, with its periodic contribution of sound practical observation and wit.

The *Dietetic and Hygienic Gazette,* to which we frequently contributed under its former management by Dr. J. C. Slay, and in which Dr. F. W. Barrows of Buffalo, has managed a nursing department, is now under the editorship of Dr. George F. Butler.

The *Homoeopathic Recorder* (Sept. 15, 1912), regards medical unity as impossible but its environment seems to be a quarter of a century behind that of western New York. For instance, while visiting a patient at a homoeopathic hospital—by no means the first we have attended there—we have met about as many old school as homoeopathic physicians and have noticed two typewritten schedules of routines to be observed in the care of patients of two men of the old school. Most of the homoeopathic physicians whom we know are members of the unspecified professional bodies.

The *Recorder* states that one of the homoeopathic firms has recently had an advertisement of a book rejected by a "regular"

lieve that the *Recorder* is right in protesting against such exclusion. We should probably regard the work as unscientific also although one of the most scientific and useful books that we have reviewed in the last half year is the work of a homoeopathic author and of a homoeopathic publishing house. But we do not believe that any man or body of men in this age of the world have the right to debar from publicity any utterance expressed in good faith. The more certain we feel that a book or paper is wrong, the less we fear that it can prevail against the truth. The fuller the opportunity for presenting fallacious reasoning, the sooner the fallacy is detected. And, the more one learns, in other words, the more scientific one becomes, the less cock-sure is he of his own absolute accuracy.

---

*The Practical Medicine* of India, Sept., 1912, quotes at length from our May issue, Dr. Little's article, "A New Departure in the Medical Expert Matter."

